# Bacterial determinants of importance in the virulence of *Gallibacterium anatis* in poultry

**DOI:** 10.1186/s13567-015-0206-z

**Published:** 2015-06-11

**Authors:** Gry Persson, Anders M Bojesen

**Affiliations:** Department of Veterinary Disease Biology, Faculty of Health Sciences, University of Copenhagen, 1870 Frederiksberg C, Denmark

## Abstract

*Gallibacterium anatis*, a member of the *Pasteurellaceae* family, constitute a part of the normal micro-flora of the upper respiratory tract and the lower genital tract in chickens. However, increasing evidence indicate that *G. anatis* is also associated with a wide range of pathological changes, particularly in the reproductive organs, which leads to decreased egg production, lowered animal welfare and increased mortality. As a recently defined opportunistic pathogen limited focus has been placed on the pathogenesis and putative virulence factors permitting *G. anatis* to cause disease. One of the most studied virulence determinants is a large RTX-like toxin (GtxA), which has been demonstrated to induce a strong leukotoxic effect on avian macrophages. A number of fimbria of different sizes and shapes has been described. Particularly fimbriae belonging to the F17-like family appears to be common in a diverse selection of *G. anatis* strains. Mutants lacking the FlfA fimbria were severely attenuated in experimentally infected chickens. Additional characteristics including the ability to express capsular material possibly involved in serum resistance; secretion of metalloproteases capable of degrading immunoglobulins, and hemagglutinins, which may promote biofilm formation are all factors likely linked to the virulence of *G. anatis*. A major advantage for the study of how *G. anatis* interact with its host is the ability to perform biologically relevant experimental infections where natural routes of exposure allows reproduction of lesions observed during spontaneous infections. This review summarizes the current understanding of the *G. anatis* pathogenesis and discusses the contribution of the established and putative virulence factors described for this bacterium to date.

## Table of contents

1. Introduction
2. Pathogenesis
  2.1 Transmission
  2.2 Experimental infections
3. Virulence factors of *Gallibacterium anatis*
  3.1 The RTX-like toxin GtxA
  3.2 Fimbriae
  3.3 Outer membrane vesicles
  3.4 Capsule
  3.5 Metalloproteases
  3.6 Biofilm formation
  3.7 Haemagglutination
  3.8 Other potential factors involved in virulence
4. Conclusions
5. Competing interests
6. Authors’ contributions
7. Acknowledgements
8. References

## 1. Introduction

**Figure 1:**
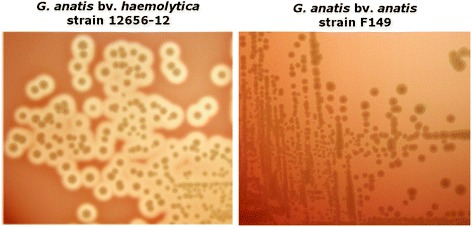
**Biovars of** ***Gallibacterium anatis*** **show difference in hemolytic properties.** Colonies of *G. anatis* biovar (bv.) *haemolytica* strain 12656–12 and *G. anatis* bv. *anatis* strain F149. Hemolysis is seen as a clearing zone around the colonies of *G. anatis* b *haemolytica*. Colonies are 1–2 mm. greyish, smooth, semitransparent, slightly raised and circular with an entire margin when incubated for 24 h at 37 °C on nutrient-rich plates containing blood. Strains of *G. anatis* biovar *haemolytica* and genomospecies 1 and 2 shows β-hemolytic zones (1–2 mm) around the colonies on agar plates with blood from calf, horse, swine, sheep, rabbit or chicken [3,10,12,13,14].

*Gallibacterium* is a genus within the *Pasteurellaceae* family [1,2] and associated with a range of avian host species. The bacterium was first described in 1950 by Kjos-Hansen as a hemolytic “cloaca bacterium” normally occurring in the cloaca of healthy chickens and cocks, but also isolated in pure cultures from numerous cases of acute salpingitis and peritonitis [3]. Since then similar bacteria, reported as *Actinobacillus salpingitidis*, avian *Pasteurella haemolytica*-like organisms or *Pasteurella anatis* were isolated and described from a number of clinical cases in chickens [4–12], before *Gallibacterium* was established as an independent genus in 2003 [2]. Colonies of *Gallibacterium* are 1–2 mm greyish, smooth, semitransparent, slightly raised and circular with an entire margin when incubated for 24 h at 37 °C on nutrient-rich plates containing blood. The genus comprises four named species; *Gallibacterium anatis*, *Gallibacterium melopsittaci* sp. nov., *Gallibacterium trehalosifermentans* sp. nov., and *Gallibacterium salpingitidis* sp. nov., and three genomospecies. *G. anatis* can be further sub-divided into two phenotypically distinct biovars; biovar *haemolytica* and the non-hemolytic biovar *anatis* (Figure 1) [2]. Strains of *G. anatis* biovar *haemolytica* and genomospecies 1 and 2 form β-hemolytic zones (1–2 mm) around the colonies on agar plates with blood from calf, horse, swine, sheep, rabbit or chicken [3,10,13,14].

*Gallibacterium anatis* is commonly isolated from chickens but has also been reported from a wide range of both domestic and non-domestic birds, including turkeys, geese, ducks, pheasants, partridges, cage birds and wild birds [1–4,15–17]. *Gallibacterium anatis* infections in humans have only very rarely been reported and here the bacterium merely seem to affect severely immuno-compromised individuals [18,19]. In the chicken, *G. anatis* is frequently found in the upper respiratory tract and lower genital tract of healthy animals. However, *G. anatis* has also been associated with a wide range of pathological lesions, especially in the reproductive organs of the egg-laying chicken and is considered a major cause of salpingitis and peritonitis in chickens, leading to lowered egg-production and increased mortality [20–22]. Moreover, *G. anatis* is globally distributed, having been isolated from poultry in countries within Europe [1,2,4,23], Africa [24], Asia [25], Australia [6] and the Americas [7,26–29].

The role of *G. anatis* as a cause of disease has been debated, yet during the more recent years increasing evidence seems to support that this organism is a likely cause of disease and lowered animal welfare. The current report aims at summarizing past and present knowledge within this area.

## 2. Pathogenesis

*Gallibacterium anatis* can be persistently isolated from the trachea and cloaca of healthy birds, showing that it constitutes a part of the normal microflora in the upper respiratory tract and lower genital tract of healthy chickens in commercial flocks [3,4,7,17,29–31]. Although *G. anatis* has been associated with a wide range of different pathological lesions, including septicemia, pericarditis, hepatitis, oophoritis, follicle degeneration, enteritis, upper respiratory tract lesions, salpingitis and peritonitis [4–12,21,24,25,27,32], the importance of *G. anatis* as a pathogen has remained controversial. No clinical picture is specifically associated with *G. anatis* and lesions cannot be distinguished from those caused by avian pathogenic *Escherichia coli* [22]. In addition, *G. anatis* is often isolated together with *E. coli* [22,33], whose importance in salpingitis is well defined [20,34,35]. However, *G. anatis* has also been isolated in pure culture from chickens suffering from different lesions [3,5,11,21,22,25,27,33,36], and a study showed that *G. anatis* was the most common single-bacterial infection in chickens with reproductive tract disorders [21], suggesting its potential as an important poultry pathogen.

Based on previous pathological findings from which *G. anatis* has been isolated, and recent investigations by Paudel et al., it seems that *G. anatis* is able to colonize the upper respiratory tract without causing clinical signs, whereas it may cause severe lesions in the reproductive tract [29,37]. This suggests a role of *G. anatis* as an opportunistic pathogen that given the right circumstances is able to cause disease. Predisposing factors such as simultaneous infection with other microorganisms [6,14,27], hormonal influences [5,11], age [4,10], seasonal changes [21], stress [38], low immunological status [39], and probably also host genetic predisposition, could explain the contradictory results obtained by experimental infection studies and bacteriological findings in naturally infected chickens.

While hens and pullets have been the main focus of previous pathogenesis research, a recent investigation by Paudel et al. [40] was aimed at elucidating the effect of experimental infections in cockerels. Interestingly, cockerels infected by intra-nasal inoculation became culture-positive in the testis and epididymis within a week post infection. The infection affected the semen quality significantly by inducing lowered sperm density, decrease in total motility and progressive motility, and reduced membrane integrity, thus clearly indicating a negative effect on fertility.

### 2.1 Transmission

The natural mode of transmission is currently being discussed. It has been widely accepted that horizontal transfer of *Gallibacterium* was the main transmission route, as the bacterium has not been isolated from chickens younger than four weeks [4,17], and that ascending infections from the cloaca appeared the most probable route to the reproductive organs [22,30]. However, one previous study showed trans-ovarian transfer at low level [41], and isolation of *G. anatis* from the egg yolk has also been observed [10,11,29,33], both indicating the possibility for vertical transmission. Moreover, quantitative (q) PCR targeting *gtxA* allowed detection of *G. anatis* in samples from chickens as young as four days [42], suggesting that the lack of sensitive methods for identification could have been the reason as to why *G. anatis* has not previously been detected in chickens younger than four weeks [4]. The fact that cockerels also seemed to get infected in the reproductive organs and semen suggests that the males potentially may play an important role in transmission between adult birds and possibly to their offspring [40]. In addition, simultaneous isolation of *G. anatis* from the trachea and internal organs of chickens suffering from salpingitis and peritonitis also indicate that given the right circumstances, the bacterium could enter the systemic circulation from its natural habitats and spread to other sites of the body [7,22,27,29,37,43]. This suggests that tissue tropism rather than a more mechanistic ascend through the oviduct, as previously anticipated, might account for the observed association of *G. anatis* with reproductive tract disorders.

The level of biosecurity seems to be important for transmission, as the bacterium is much more prevalent in production systems with low biosecurity level [30]. So, although horizontal transfer may be the main mode of transmission within a flock, it appears plausible that vertical transfer may be an important link between flocks.

### 2.2 Experimental infections

The circumstances that shift *G. anatis* from being a normal inhabitant of the mucosal surfaces to a pathogen causing severe lesions are not known. Most early experimental infection studies aimed at elucidating the exact role of *G. anatis* as a pathogen have not allowed firm conclusions due to varying study designs and use of different strains. The results ranged from no obvious pathogenicity [4,17,32] to disease resembling the natural infection including re-isolation of bacteria from multiple organs, reduced egg-production and increased mortality [3,8,10,11,14,29,44–46].

As previously discussed, it is possible that the presence of predisposing factors, such as impaired immunity, is necessary for establishment of a consistent clinical picture of experimental infections. The opportunistic potential of *G. anatis* was shown by Bojesen et al., who demonstrated that experimental infection of 15-week old heterophil-depleted chickens resulted in lesions similar to those induced during natural infections from which *G. anatis* had been isolated, whereas non-depleted chickens showed less pronounced lesions [39].

Other important factors that probably play a significant role for the contradictory results are the difference in virulence between strains and the uncertain identification and classification of the organism in the early studies [4,5,37,43,47]. The pronounced genetic diversity may thus be reflected in differences in the possession and expression of different virulence factors [48,49].

An alternative explanation to the different results could also lie in the applied in vivo infection model. It seems obvious that depending on the aim of the investigations, it is crucial to use the most biologically relevant model. *Gallibacterium anatis* is an inhabitant of the upper respiratory tract and lower reproductive tract mucosa in the chicken. Thus intramuscular, sub-cutaneous or intravenous injections of *Gallibacterium* might not give a realistic picture of the natural course of the infection. Recent studies using intra-nasal instillation of specific pathogen free (SPF) chickens have shown that the bacteria were able to spread to internal organs, most particular colonizing organs of the reproductive tract, resulting in lesions similar to natural infections [29,37]. Intra-nasal instillation could be a good model for studying colonization of the airway [37,43]. However, by using intra-nasal instillation it might be difficult to quantify the exact number of bacteria that actually enter the host. This also appears to be reflected in the varying degree of re-isolation of *G. anatis*, even with the same strain [37], which makes it difficult to evaluate and compare the exact virulence of different strains of *G. anatis*. Moreover, as *G. anatis* is considered an opportunistic bacterium, the simultaneous presence of other bacterial species could have an unidentified impact on the pathogenesis of *Gallibacterium*. Vazquez et al. showed that experimental infection with *G. anatis* had a more severe effect on egg production in commercial chickens than SPF chickens [46].

Several studies point at *G. anatis* being particularly involved in infections of the reproductive tract and peritoneum [3,46] and this view has been supported by Mirle et al. who showed that *G. anatis* was among the most common bacterial agents isolated from lesions in the reproductive organs [21] during natural infections. Therefore, to study the pathogenic nature of the bacterium within these organs it would be feasible to use a model specifically targeting these organs. Intra-peritoneal injection bypasses some of the early stages of the immune response and has been used in numerous cases to study the virulence of *Gallibacterium* [3,4,10,11,14,32,39]. This model may imitate a natural infection of the peritoneum, while one of the draw-backs is that the bacteria quickly enters systemic circulation and thus might affect multiple organs not related to the natural course of infection, giving an inaccurate and complicated clinical picture that might make it more difficult to control the course of disease. Recently, a new animal model has enabled experimental infections of the oviduct, in which an avian pathogenic strain of *E. coli* are injected into the lumen of the oviduct by a simple surgical procedure [50]. Furthermore, the controlled deposition of the inoculum makes is easier to control and study the infection. The inoculation with *E. coli* resulted in peritonitis, salpingitis, oophoritis and necrotic hepatitis – all in line with the clinical picture seen during the natural course of infection with this strain [50]. Thus, this in vivo model might resemble the natural infection of the reproductive tract more than the intra-peritoneal infection model.

Using an appropriate infection model to study virulence factors and host-pathogen interaction could lead us to a deeper understanding of the pathogenic nature of *G. anatis*, and thus aid in the development of new prevention and treatment strategies.

## 3. Virulence factors of *Gallibacterium anatis*

Virulence factors are defined as components of an organism that give it the ability to cause disease, and thus determine the pathogenicity of the organisms, but are dispensable for its viability. Virulence factors are involved in many aspects of the host-pathogen interface, including colonization, nutrient acquisition, immune-evasion and immunosuppression, and include toxins, enzymes and adhesion molecules. Limited knowledge has been obtained about the mechanisms involved in the pathogenesis of *G. anatis* and few virulence factors have been characterized in depth.

### 3.1 The RTX-like toxin GtxA

One of the main characteristics used for identification of *G. anatis* biovar *haemolytica* is its ability on blood-agar plates to form a broad β-hemolytic zone around the colony. The protein responsible is the secreted toxin named GtxA (*Gallibacterium* toxin A), which is the most well described virulence factor of *G. anatis* [13,48]. The GtxA protein expressed by *G. anatis* possesses hemolytic activity against erythrocytes from a wide variety of hosts, and leukotoxic activity against the chicken macrophage cell line HD11 [13].

**Figure 2:**
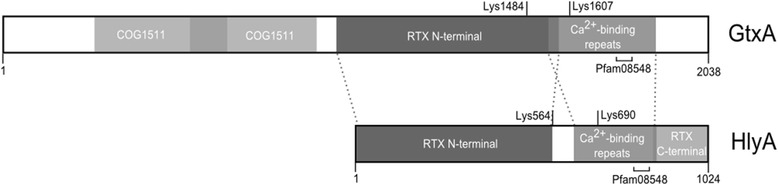
**GtxA is a novel RTX-like protein with an unusual domain organization.** Schematic presentation of the domain organization of GtxA from *G. anatis* biovar *haemolytica* strain 12656–12 [RefSeq:WP_013746567] compared to the typical domain organization of RTX toxins represented by HlyA from *E. coli* [PRF:225074]. HlyA has 34% coverage and 29% identity with GtxA. Regions of homology are shown with dotted lines. The GtxA toxin is comprised of 2038 amino acids (aa). In the N-terminal part of GtxA (approximately aa 1 – 950), two overlapping regions with weak homology to a membrane protein with unknown function (COG1511) is present. The remaining C-terminal part of GtxA has homology to HlyA by having a RTX N-terminal domain (pfam02382), the Ca^2+^-binding repeats (COG2931) and a domain classified as peptidase M10 serralysin C terminal (pfam08548). GtxA also contains the lysine residues, Lys1484 and Lys1607, required for activation of the toxin by acetylation by GtxC. The homologue lysine residues in HlyA are found at Lys564 and Lys690. In addition to the described domains, HlyA also contains a RTX C-terminal domain (pfam08339). Domains were identified using NCBI conserved domain search with default settings.

RTX-toxins are pore forming exoproteins that are secreted via a type I secretion system (T1SS) in Gram-negative bacteria. Their name originates from a region, generally found in the carboxyl terminal of the protein, which contains a series of Ca^2+^ binding glycine-rich nonapeptide repeats (repeats in the structural toxin) [51,52]. RTX toxins are expressed by many members of *Pasteurellaceae* where they are responsible for the hemolytic and also the leukotoxic phenotype of these bacteria [51]. The RTX toxins are usually transcribed from a four-gene operon, comprising the genes *rtxC*, *rtxA*, *rtxB* and *rtxD* in transcribed order, respectively. The gene *rtxC* encodes an activation protein that acetylates the toxin, encoded by *rtxA*. The genes *rtxB* and *rtxD* encode translocator proteins that together with the outer membrane protein named TolC comprise the T1SS for the functional RTX toxin. The homologue toxin, HlyA, from *E. coli* is often used as the model for describing the typical RTX toxin, although variation across species does exist. A schematic presentation of HlyA and GtxA domain organization is shown in Figure 2. GtxA is unusually large; with its 2038 amino acids it is almost twice the size of HlyA. It consists of two domains: a C-terminal domain with homology to other RTX toxins responsible for the hemolytic function; and a N-terminal domain of approximately 950 amino acids with unknown function and no obvious homologs, but which is required for full hemolytic activity and the leukotoxic activity of the toxin (Figure 2) [13]. A similar structure is seen for the recently identified 238 kDa RTX-like toxin named AvxA from *Avibacterium paragallinarum*, in which only the C-terminal part of the protein show homology to RTX toxins, while the N-terminal function as a serine protease [53]. Moreover, at the genetic level an atypical organization for *gtxA* is seen, while *gtxA* and *gtxC* is located together, the genes *gtxB*, −*D* and the *E. coli tolC* homologue *gtxE* are located elsewhere in the genome [13].

The role of GtxA in the pathogenesis is not fully understood, although a *gtxA* knockout mutant is clearly attenuated in virulence (Pors, S., unpublished data). The hemolytic effect seems unspecific as hemolysis is observed against a wide range of blood cells from different animal species [3,23,54], indicating that lysis of erythrocytes might not be the main target for GtxA. The N-terminus contains a domain with weak homology to Talin, a protein involved in the linkage of the cytoplasmic portion of integrins to the actin cytoskeleton by interactions with vinculin and alpha-actinin [55,56]. Actin plays many physiologically important roles in the cell, some of which also involve the regulation of immune cell recognition and adherence, production and release of immune cell signaling molecules and phagocytosis. It is therefore not surprising that several classes of bacterial toxins target the actin cytoskeleton of the host cells as an immune evasion strategy [57]. Some high molecular weight toxins belonging to the RTX family, called MARTX (multifunctional autoprocessing RTX), have been show to bind to and modulate actin [57]. It could therefore be speculated that GtxA represents a novel form of RTX-like toxin, with a proposed function in immune evasion.

### 3.2 Fimbriae

**Figure 3:**
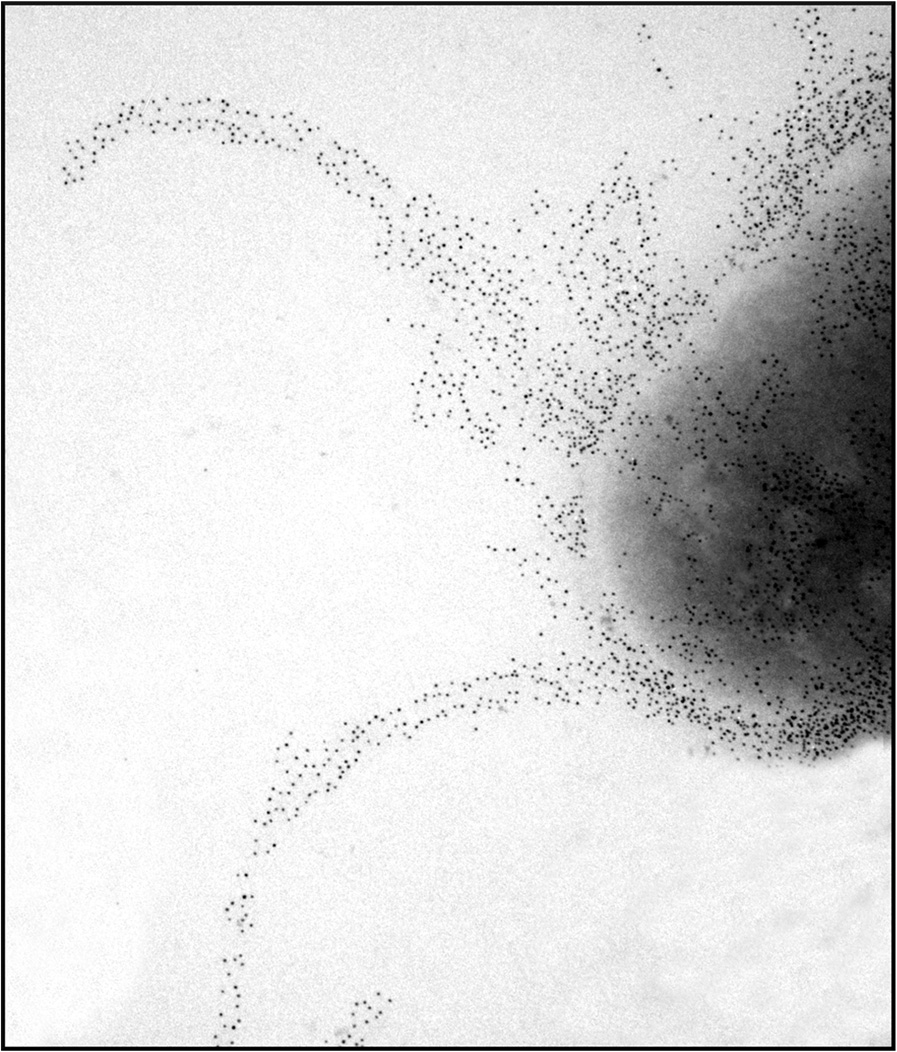
**The F17-like fimbria, FlfA, is exposed on the surface of** ***G. anatis*** **.** FlfA on the surface of *G. anatis* strain 12656–12 is shown by immunogold electron microscopy using anti-FlfA serum, followed by a secondary antibody conjugated to 10-nm gold particles. Picture modified from Copyright © American Society for Microbiology, [63].

An important aspect of colonization is the ability to adhere to and invade host tissue. It has been shown that *G. anatis* has the ability to adhere to chicken epithelial cells [58] and inert surfaces, and short fimbria-like structures on the bacteria have been observed [59]. Fimbriae are hair-like structures expanding from the surface of the bacteria, and are often involved in adhesion to host cells [60]. Recently, several F-17 like fimbriae clusters were identified in the genomes of three different *G. anatis* strains [49]. F-17 like fimbriae belong to a group of fimbriae that bind N-acetyl-D-glucosamine (Glc-NAc) containing receptors on the surface of host cells, and is thus, thought to be involved in adhesion of the bacteria to the mucosal surfaces within the host [61]. This type of fimbria is expressed by several pathogenic types of *Escherichia coli*, including strains of avian pathogenic *E. coli* (APEC) [61,62]. In *G. anatis* the F17-like fimbria is encoded by a four-gene cluster comprising a gene encoding a chaperone, an usher protein, an adhesion protein and a structural subunit protein designated as *flfD*, *flfC*, *flfG* and *flfA*, respectively [63]. The chaperone and the usher protein facilitate folding, assembly and secretion of the structural subunit protein, which make up the stem of the fimbriae. The adhesin is located at the tip of the fimbrial structure and is responsible for receptor recognition and binding [61,64]. A recent comprehensive study of the fimbriae genes in the genome of 22 *G. anatis* strains showed that F17-like fimbriae are very common with strains encoding between 1–3 different fimbrial clusters [65]. Based on the structural genes, these clusters could be divided into five phylogenetic types (Flf, Flf1-4), with the cluster designated Flf1 being the most frequently occurring fimbrial cluster present in 74% of the genomes investigated. Interestingly, none of the strains encoding Flf1 appeared to express the structural protein, FlfA1, in vitro. In contrast, the Flf cluster, encoding the previously described 20.5 kDa fimbrial subunit protein FlfA (Figure 3) [63], was encoded in 65% of the strains and expressed in vitro by 79% of these strains [65].

The genetic variation was greatest in the genes encoding the structural proteins and the adhesin. Polyclonal antibodies raised against the three most common types of the structural subunit proteins did not cross-react with the other Flf-types [65]. This indicates that the structural subunit and the adhesin are under selective pressure for immune recognition, and thereby could indicate the importance of fimbriae during the pathogenesis of *G. anatis*. This has been supported by Bager et al., who showed that the FlfA fimbria is important for virulence in vivo, as a knockout mutant of *flfA* was attenuated. From this it was also suggested that fimbrial expression may govern the tissue tropism observed for *G. anatis* [22,63]. The presence of several fimbrial clusters could be a result of an immunogenic pressure favoring duplication events and increased affinity for different targets in the host tissue, or could reflect a functional diversity, where different fimbria are expressed at different time points during infection. The exact role, target cells and regulation of the identified fimbriae in *G. anatis* still need to be determined.

### 3.3 Outer membrane vesicles

**Figure 4:**
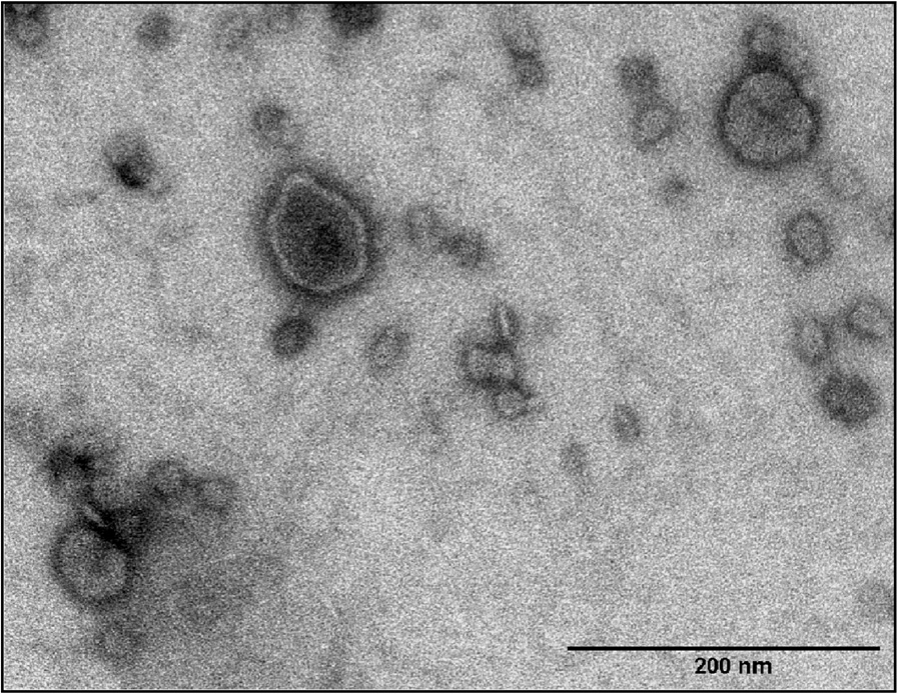
**Outer membrane vesicles (OMVs) are naturally secreted from** ***G. anatis*** **.** OMVs are seen as spherical structures in sizes ranging from 20 nm and up to 160 nm. The OMVs were isolated from *G. anatis* strain 12656–12 and viewed by transmission electron microscopy with 2% (w/v) phosphotungstic acid staining [69].

Outer membrane vesicles (OMV) are spherical, bilayered membrane structures, which have been associated with an enormous functional diversity. OMVs are released by virtually all Gram-negative bacteria where they are produced by budding of the outer membrane and therefore mainly consist of outer membrane components, such as membrane associated proteins and LPS, although, they have also been shown to contain periplasmic components and even compounds of cytoplasmic origin such as DNA [66–68]. Recently it was demonstrated that *G. anatis* produce OMVs (Figure 4) with a protein content that varied depending on growth condition [69], suggesting that OMV production could serve multiple functions and be a way to cope with changing environments, e.g. within the host. To study the formation of OMVs, an OMV-overproducing mutant of *G. anatis* was created by knocking out the gene *tolR*, encoding a protein within the Tol-Pal system, which is crucial for membrane stability and integrity [69,70]. The mutant released considerably higher amounts of OMVs, supporting that membrane stability is essential for OMV formation [69].

The functions of *Gallibacterium* OMVs have not been determined. OMVs could act as a means to get rid of misfolded or excess proteins or enable replacement of lipids in the outer membrane during growth. However, the presence of specific molecules, e.g. proteins, lipids or polysaccharides, under different circumstances indicates an active role of the OMVs. In other species OMVs have been ascribed importance for biofilm formation [71] and for carrying quorum sensing molecules involved in cell-to-cell communication [72]. Microvesicles produced by *G. anatis* adhering to glass has previously been observed [59]. However, increased release of OMVs by the *tolR* knockout mutant does not seem to have any effect on the level of biofilm formation (unpublished data). The Δ*tolR* mutant OMVs are much more uniform and less affected by environmental changes compared to wild-type OMVs [69]. This could be due to the more artificial nature of the mutant OMVs, which may lead to a lack of specific molecules within the vesicles, e.g. factors required for biofilm formation. OMVs have also been shown to act as transportation vehicles for the delivery of lipids, membrane proteins, insoluble compounds or compounds that are easily degraded, including toxins and DNA. The transport of proteases and hemolysins by OMVs has been shown for *Actinobacillus pleuropneumoniae* and *Avibacterium paragallinarum* [73,74]. However, vesicles from *G. anatis* do not seem to be involved in either hemolysis or proteolysis (unpublished data). A few proteins have been identified as being a part of the OMVs, including a possible hemagglutinin with sequence similarity to the filamentous hemagglutinin protein precursor FhaB from *Bordetella pertussis*, which has been found important for colonization of the host mucosa [69,75]. Another OMV associated protein in *G. anatis* is MDN1, an AAA ATPase containing a vWA domain, which in other species has been suggested to be important for the bacterial stress response, cell adhesion and/or biofilm formation [69,76]. From this, it could be speculated that OMVs from *G. anatis* could act as a possible virulence factor important for adherence and colonization of the host. Other possible functions of OMVs include modulating the host immune response, acting as target for phages or being involved in the binding and removal of anti-bacterial substances including different antibiotics, all aiding in the survival of the microorganism [67,68]. Despite the many speculations, the role of *G*. anatis OMVs has not been determined. As they seem affected by the surroundings, it is likely that additional host factors, such as those found in serum, might play a role in regulation and function of the OMVs. The amount of OMVs produced as well as the protein profile dramatically changes when *G. anatis* is incubated in the presence of serum (unpublished data), supporting the hypothesis that the OMVs might play a role in the bacterium’s interaction with its natural environment, the chicken host.

### 3.4 Capsule

**Figure 5:**
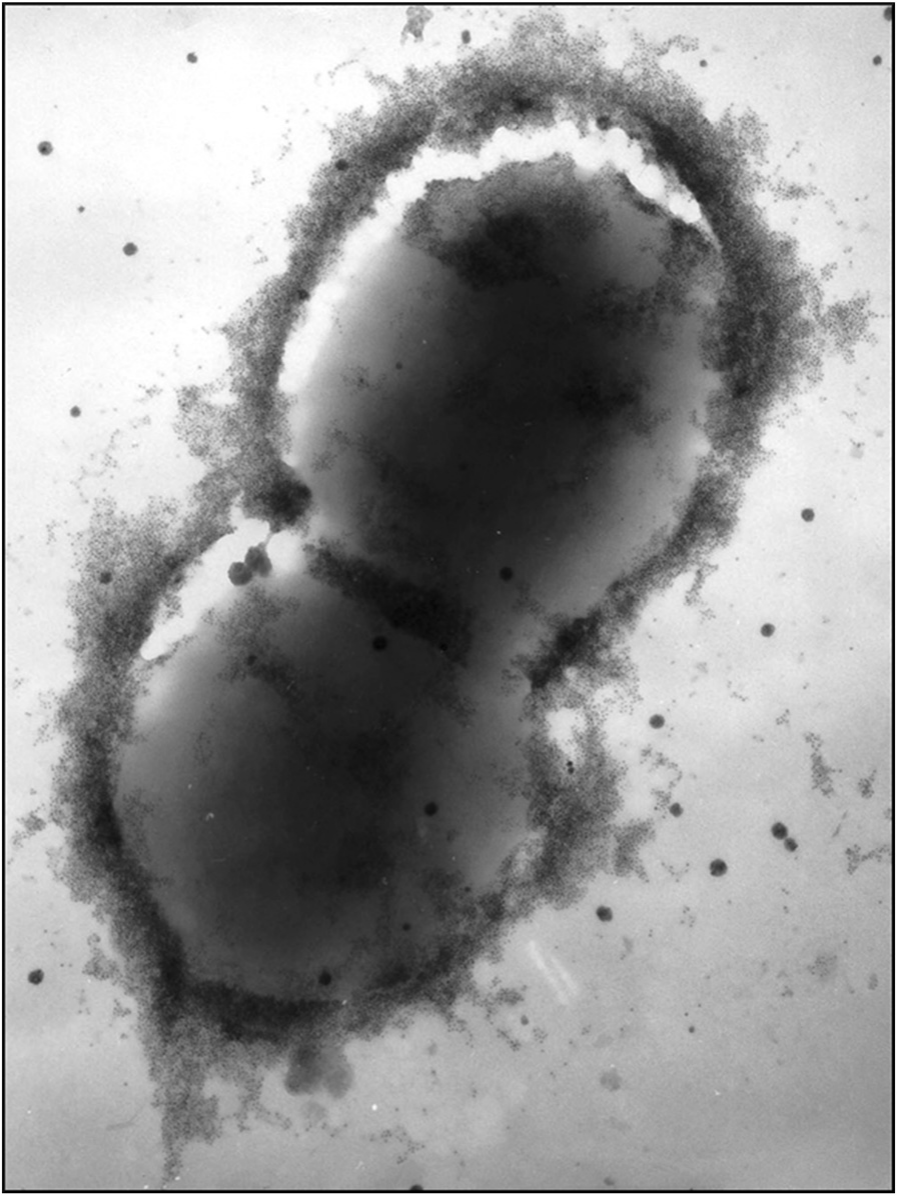
**Capsular material is present on the surface of** ***G. anatis*** **.** A thin capsular structure can be seen on the surface of *G. anatis* strain 12656–12 using uranyl acetate staining followed by transmission electron microscopy [80].

Bacterial capsules are composed of extracellular polysaccharide, and are found in a diverse array of both Gram-negative and Gram-positive pathogens [77]. Functional importance has been documented in relation to adhesion, cell-cell interactions and immune evasion [78,79]. The presence of a thin capsule on *G. anatis* has been observed by transmission electron microscopy (Figure 5) [80] and Kjos-Hansen observed the presence of a capsule in primary culture, which however, disappeared after sub-cultivation. Moreover, the capsule was not present in isolates from healthy chickens [3]. Analysis of the genomes of three *G. anatis* strains revealed a capsular locus [49] in one of the strains. The function of the capsule of *Gallibacterium* is not known. Interestingly, a capsule-knockout mutant (Δ*gexD*) proved to be more virulent than its wild-type counterpart [80]. Although further studies are needed to elucidate this, it could be speculated that removal of the capsule led to the exposure of some normally hidden antigens or pathogen-associated molecular patterns (PAMPs), resulting in an increased immune response against *G. anatis*.

### 3.5 Metalloproteases

Proteases are enzymes that catalyze the hydrolysis of peptide bonds in proteins or peptides. These enzymes seem to catalyze many essential functions of pathogenic bacteria. Metalloproteases are one class of proteases that play many important functions in virulence, including colonization, nutrient acquisition, immune evasion, and bacterial invasion into the systemic circulation [81]. Modulation of the host immune response can be facilitated by e.g. acting on serum components, such as immunoglobulins and proteins of the complement system [81]. *G. anatis* expresses metalloproteases capable of degrading avian immunoglobulin IgG [82] suggesting a possible role in immune evasion. Although, the genetics behind this function have not been determined, several metalloproteases are encoded within the genome of *G. anatis*, including an extracellular protein with a metal-dependent endonuclease domain, a zinc metalloprotease and an ATP-dependent metalloprotease [49]. One or more of these proteins could be responsible for the proteolytic capability of *G. anatis*. The specificity or role in pathogenesis of the IgG-degrading metalloproteases is not known, but it has been speculated that this protein could be responsible for the host-specific pathogenicity observed for some strains [43].

### 3.6 Biofilm formation

Bacterial biofilms are structured cell communities embedded in a polymeric matrix that allows adherence to surfaces and live tissue. Clinically, biofilm formation is associated with persistent and chronic infections and increased resistance to antimicrobials [83,84]. It has been shown that *G. anatis* is capable of binding to inert surfaces, which is regarded as a first step towards biofilm formation [59]. The ability to form biofilm varies between isolates, and Johnson et al. was able to divide strains of *G. anatis* into groups of weak, moderate and strong biofilm producers [49]. Although no clear correlation between formation of biofilm and pathogenesis could be noted [59], this grouping of strains according to the level of biofilm formed, revealed a pattern corresponding to the apparent evolutionary decent of the strains [49]. This could indicate that biofilm formation might play an important role for certain clades within *G. anatis*.

### 3.7 Hemagglutination

Some strains of *G. anatis* have been found capable of agglutinating avian erythrocytes [85,86]. Hemagglutination is linked with the expression of hemagglutinins or adhesins capable of binding receptors on the surface of red blood cells. Genome analysis revealed the presence of a number of putative hemagglutinins, and the presence of a potential hemagglutinin in OMVs released from *G. anatis* has been observed [49,69]. Some of these hemagglutinins could be responsible for the observed agglutinating activity seen for some strains [49,69,87]. The role and importance of these proposed hemagglutinins has not been determined, yet hemagglutinating activity have been shown to be important for other poultry pathogens, such as *Avibacterium paragallinarum* [88,89].

### 3.8 Other potential factors involved in virulence

Sequencing and subsequent analysis of the genomes of *G. anatis* will undoubtedly reveal many more potential virulence factors in the future. Analysis of three *G. anatis* genomes identified several so-called clustered regularly interspaced short palindromic repeats (CRISPR) in all strains, some of which were shared with other strains, while others where strain specific [49]. CRISPRs function as a bacterial defense system protecting the bacterium against foreign invasive DNA, such as DNA from phages and plasmids, and can be thought of as a bacterial immune system. CRISPR have been shown to be able to interfere with transformation [90], which could explain the difference in natural competence seen between strains of *G. anatis* [91].

The natural competence induced in *G. anatis* when nutrients are scarce could, may be a way for the bacteria to exchange genes, encoding virulence factors and other genetic elements important for colonization of the host, eventually leading to a better chance of survival. Integrative conjugative elements (ICE) are elements that are able to excise and integrate in the genome by genes encoded within these elements [92]. ICEs have recently been identified in the genomes of *G. anatis* [49]. Often, these elements carry antimicrobial resistance genes. Although antimicrobial resistance is widespread among isolates of *G. anatis* [93], the ICEs did not seem to contain any antimicrobial resistance genes. The presence of mobile elements adjacent to genes encoding fimbrial clusters (*flf*) have also been identified [65]. This could allow the spread of different fimbrial types between strains of *G. anatis*. The uptake of plasmids containing antimicrobial resistance and virulence factors could also be important for the emergence of virulent clones. Strains of *G. anatis* contain up to four plasmids of varying sizes [2]. The plasmids are largely un-characterized and the presence of antimicrobial resistance genes carried by the plasmids have not been demonstrated [49]. It is likely that since resistance towards some antimicrobials are so conserved, the resistance might be encoded chromosomally. Although antimicrobial resistance cannot be directly stated as a virulence factor, the spread of resistance genes located on mobile elements, such as those described above, might lead to the co-mobilization of virulence factors and thus increase the overall pathogenicity of *G. anatis* [94].

Early studies have described the presence of small colony variants (SCVs), especially observed in primary cultures of *Gallibacterium* [8,10,12], some of which show differences in hemolytic activity [12]. SCVs are associated with increased persistence, recurrent infections and increased resistance towards antimicrobials [95]. Apart from the early findings, the ability of *G. anatis* to form SCVs is unexplored and the importance of this phenotype in pathogenesis and treatment remains to be studied.

Several aspects concerning the contribution of each individual virulence factor in the pathogenesis of *G*. anatis remain to be determined. Yet, further genome sequence analysis and characterization of potential disease determinants in the natural host will likely contribute to the elucidation of *G. anatis* as a pathogen.

## 4. Conclusions

The involvement of *G. anatis* in reproductive tract disorders in chickens poses a serious economic and welfare problem in poultry production. Yet, the circumstances determining whether *G. anatis* remains a peaceful part of the mucosal microflora or changes into a disease causing pathogen largely remains to be addressed.

The development of diagnostic kits that simultaneously allow identification of other pathogens commonly associated with reproductive tract disorders in chickens could help to clarify the role and co-existence of the most common pathogens (e.g. *E. coli*) in this organ system. Identification of optimal growth media, determination of antibiotic resistance markers, assessment of virulence factors and possible vaccine candidate proteins by whole genome sequence analysis would further aid in the process of developing new treatment or prevention strategies.

A deeper insight into the basic biology of *G. anatis* is also likely to improve the understanding of this organism. Many of the early papers indicate that the bacteria grow best under micro-aerophillic conditions, and have reported different size variants with different morphology and biochemical properties [3,12]. A standardized cultivation scheme does not necessarily take this into account, and the repeated sub-cultivation of laboratory strains might lead to the loss of expression of virulence factors initially important for the strains when originally isolated from diseased chickens. One evident example of this is the loss of capsule when sub-cultivating primary isolates [3]. In order to be able to understand the exact host-pathogen interplay, and the switch from a normal inhabitant of the healthy chicken to a pathogen causing disease and mortality, further insight into the expression of virulence factors under more biologically relevant circumstances is desirable.
